# Genetic Insights: Balancing Milk Yield, Fat: Protein Ratio and Fertility in Primiparous Cows From Subtropical Regions

**DOI:** 10.1111/jbg.12944

**Published:** 2025-05-30

**Authors:** Amauri Felipe Evangelista, Altair Antônio Valloto, Lenira El Faro, Rodrigo Junqueira Pereira, Laila Talarico Dias, Rodrigo de Almeida Teixeira

**Affiliations:** ^1^ Department of Animal Science Federal University of Paraná Curitiba Brazil; ^2^ Holstein Cattle Breeders Association of Paraná Curitiba Brazil; ^3^ Institute of Animal Science Advanced Beef Cattle Research Center Sertãozinho Brazil; ^4^ Institute of Agricultural and Technological Sciences Institute of Agricultural and Technological Sciences, Federal University of Rondonópolis Mato Grosso Brazil

**Keywords:** calving interval, correlated response, days open, energy balance

## Abstract

In this study, we aimed to evaluate the genetic association between fertility traits, milk yield and the fat: protein ratio (FPR) on the test day in primiparous Holstein cows. The analysed traits were milk yield (TDMY) and FPR assessed on the test day, as well as the following fertility traits: period from calving to first service (CFS), days open (DO) and calving interval (CI). Genetic parameters were estimated through bivariate analysis, using a random regression model (considering fourth‐order Legendre polynomials) and the Bayesian method, with GIBBS2F90 software. Heritability estimates varied between 0.11 and 0.22 for TDMY, between 0.16 and 0.30 for FPR and between 0.03 and 0.05 for the fertility traits. Correlation estimates between TDMY and fertility traits tended to increase from early lactation until approximately day 100, then decreased slightly before continuing to grow until the end of lactation. Genetic correlations between TDMY and FPR were negative throughout lactation, ranging from −0.04 on day 5 to −0.37 in the final third of this period. The genetic correlations between FPR and fertility traits were positive in early lactation and negative in late lactation (except for CFS). These results indicate that TDMY and FPR are heritable and can be used as selection criteria in Holstein cows in Brazil. However, for fertility traits, genetic gains through direct selection may be slow. Additionally, a high level of milk production and FPRs in early lactation negatively impact fertility traits.

## 1 Introduction

Genetic improvement is an important tool for enhancing the productivity indices of dairy herds. However, selection practices focused on increasing milk, fat and protein yields have led to declining fertility (Lima et al. 2020). In addition to reducing reproductive lifespan, longer calving intervals (CIs) and higher culling rates due to reproductive failures are two major issues contributing to production losses (de Vries and Marcondes 2020).

Consequently, diminished fertility‐related performance not only reduces genetic gains in production traits but also escalates insemination costs, leading to higher rates of involuntary culling and reduced milk yield per animal (Sathwara et al. 2020). In their study of dairy cow herds in Thailand, Buaban (2016) identified an unfavourable genetic correlation between test‐day milk yield (TDMY) and fertility traits. The researchers concluded that selection for higher milk yield, particularly in early lactation, markedly prolongs the duration to first service and is associated with extended periods of days open (DO) and longer CIs.

Satola and Ptak (2019) observed that high‐producing dairy cows tend to exhibit poorer fertility performance and a higher incidence of health disorders, leading to a more pronounced negative energy balance (NEB) during early lactation. Similarly, Soares et al. (2021) reported that cows unable to adequately adapt to NEB are more prone to metabolic disorders, a widespread issue in dairy farming that can result in substantial economic losses. The milk fat: protein ratio (FPR) has been proposed as a practical indicator of energy balance in early lactation due to its ease of measurement, low cost and effectiveness in predicting fertility‐related problems and metabolic disorders in dairy herds (Jamrozik et al. 2016; Ranaraja et al. 2018). According to Kaoian (2023), an FPR greater than 1.5 denotes a large energy deficit in dairy cows.

Buttchereit (2010) estimated a significant and negative genetic correlation (ranging from −0.43 to −0.42) between FPR and energy balance during early lactation. They concluded that FPR serves as a valuable marker of energy balance, particularly in early lactation, the most critical period for dairy cows. Furthermore, estimating genetic variations in traits indicative of energy balance and their genetic correlations with fertility traits may facilitate the development of innovative breeding and management strategies to enhance efficiency, health and fertility in dairy cows (Mehtio et al. 2020).

Therefore, the identification of robust animals less susceptible to variations in NEB requires pinpointing traits measurable during the early stages of a cow's productive life that are routinely included in milk testing practices on farms. In this context, our study aims to evaluate genetic associations between fertility traits, milk yield and FPR in primiparous Holstein cows.

## 2 Materials and Methods

### 2.1 Data and Editing

Data from the Milk Testing Service of the Holstein Cattle Breeders Association of Paraná (APCBRH), located in Curitiba, Paraná, southern Brazil, were utilised. The dataset included animals from 343 herds across the southern region of the country, with lactation records spanning from 2010 to 2017. Monthly data on TDMY and fat and protein percentages from first‐lactation cows were analysed. The ages of the animals at calving ranged from 18 to 43 months, and they were monitored from the 5th day after calving until the 305th day in milk. The FPR was calculated as the simple ratio of the fat percentage to the protein percentage in the milk.

TDMY and FPR were classified into fortnightly day‐in‐milk classes, totaling 20 classes throughout the period; for example, from 5 to 20, 21–35, 36–50 days and so forth, until reaching 305 days. Additionally, the following fertility traits were included: period from calving to first service (CFS), defined as the number of days from calving to the first insemination; DO, considered the number of days from calving to the last insemination; and CI, that is, the number of days between the first and second calvings.

Records were excluded if they belonged to herds with fewer than 10 animals, cows with fewer than five test days during lactation, cows whose first test was conducted more than 45 days after calving, or cows with a daily milk yield below 5 kg or above 80 kg. Only cows milked two or three times a day were considered in the evaluation. Consistency for FPR was verified through graphical inspection, ensuring observations ranged between 0.35 and 1.90.

The contemporary group (CG) for TDMY and FPR was defined by herd, year and month of milk testing, while for fertility traits, the CG was defined by herd, year and month of calving. Records with yields higher or lower than 3.5 standard deviations within the CG, as well as CGs with fewer than three cows, were excluded from the analysis. For TDMY and FPR, records were segmented into subclasses formed by Herd‐Year of Calving, each containing at least five animals. This effect was included because it would be responsible for modelling the differences in persistency/shape of the lactation curve among the herds over time (Jamrozik et al. 2001; de Roos 2004).

The dataset comprised 81,272 animals, including 2437 bulls and 45,356 dams in the relationship matrix. Table 1 presents the descriptive statistics for the studied variables.

**TABLE 1:** Descriptive statistics for production and fertility traits of Holstein cows in southern Brazil.

Trait	No. of animals	Tests	Mean ± SD	Min.	Max	No. of CGs
TDMY (kg)	47,215	414,356	31.20 ± 7.42	5	76	17,010
FPR	47,265	413,800	1.10 ± 0.21	0.35	1.90	17,012
CFS (days)	26,885	—	93.95 ± 46.97	20	360	4616
DO (days)	27,031	—	143.22 ± 80.29	23	400	4632
CI (days)	28,563	—	421.33 ± 81.17	300	680	4820

Abbreviations: CFS = period from calving to first service, CG = contemporary groups, CI = calving interval, DO = days open, FPR = fat: protein ratio, SD = standard deviation, TDMY = test‐day milk yield.

### 2.2 Genetic Analysis

Bivariate analyses between TDMY and FPR, as well as between TDMY, FPR and fertility traits (CFS, DO and CI), were performed using a random regression model. Due to the absence of repeated measures for the fertility traits, only the intercept of the additive genetic effect was modelled, along with the corresponding fixed effects.

The random regression model employed can be described as:

$$\begin{array}{c} y_{\text{ijkl}}={\mathrm{CG}}_{i}+{\mathrm{FC}}_{j}+\sum_{m=0}^{3}{\mathrm{RA}}_{\mathrm{km}}\Phi_{m}\left(d\right)+\sum_{m=0}^{3}\alpha_{\mathrm{lm}}\Phi_{m}\left(d\right)\\ +\sum_{m=0}^{3}\rho_{\mathrm{lm}}\Phi_{m}\left(d\right)+E_{\text{ijkl}} \end{array}$$

where $y_{\text{ijkl}}$ is the observed value of the trait; ${\mathrm{CG}}_{i}$ is the fixed effect of CG *i*; ${\mathrm{FC}}_{j}$ is the fixed effect of milking frequency *j* (two or three times) nested within the days‐in‐milk class (20 classes); ${\mathrm{RA}}_{\mathrm{km}}$ is regression coefficient m (fixed effect), specific to the herd subclass–year of calving *k*; $\alpha_{\mathrm{lm}}$ and $\rho_{\mathrm{lm}}$ are regression coefficients *m* for the additive genetic and permanent environment random effects of cow *l*, respectively; $\Phi_{m}\left(d\right)$ is the *m*‐th order orthogonal Legendre polynomial corresponding to day in milk *k*; and $E_{\text{ijkl}}$ is the random error associated with the observation. Fourth‐order (cubic) Legendre polynomials were used for the TDMY and FPR regressions. Different polynomial orders were previously tested to determine which would best explain the trajectories of (co)variances for the additive genetic and permanent environmental effects of the animal.

The model can be represented in matrix form by:

y=Xβ+Za+Wp+e

where y is the vector of observations; β is the vector of fixed effects; a is the vector of solutions for the direct additive genetic random regression coefficients; p is the vector of solutions for the permanent environment random regression coefficients; X, Z and W are the incidence matrices of β, a and p, respectively, and e is the vector of residuals.

For the fertility traits (CFS, CI and DO), the animal model included the systematic effects of the CG (herd‐year‐month of calving), age of the cow at calving (linear and quadratic effects) and the additive genetic and residual random effects. Moreover, for these traits, a permanent environmental effect was included in the model to ensure that the covariance between environmental effects was attributed to the covariance between permanent environmental effects rather than residual effects when one of the traits was measured more than once (Hanford et al. 2003). Thus, the environmental variance for the fertility trait was calculated by summing the variance components for permanent and residual environmental effects.

For the fertility traits, the model can be represented in matrix form by:

y=Xβ+Za+e

where y is the vector of observations; β is the vector of fixed effects; a is the vector of solutions for the direct additive genetic random regression coefficients; X and W are the incidence matrices of β and a, respectively, and e is the vector of residuals.

To obtain the genetic correlations between FPR and TDMY measured at different days in milk (DIM: every 15 days from 20 to 305) and fertility traits (CFS, CI and DO—Fert), the random regression model was extended to a bivariate analysis, as described by Veerkamp et al. (2001), in which the (co)variance matrix was obtained as V M V', where M is the estimated covariance matrix and *V*, is the matrix of legendre polynomials evaluated at each DIM, defined as follows:

The general structure of matrix *M* is given by:

$$M=\left[\begin{array}{ccccc} \sigma_{\text{Fert}}^{2} & \text{σFert},\beta_{0} & \text{σFert},\beta_{1} & \text{σFert},\beta_{2} & \text{σFert},\beta_{3}\\ . & \sigma_{\beta 0}^{2} & {\sigma \beta }_{0},\beta_{1} & {\sigma \beta }_{0},\beta_{2} & {\sigma \beta }_{0},\beta_{3}\\ . & . & \sigma_{\beta 1}^{2} & {\sigma \beta }_{1},\beta_{2} & {\sigma \beta }_{1},\beta_{3}\\ . & . & . & \sigma_{\beta 2}^{2} & {\sigma \beta }_{2},\beta_{3}\\ . & . & . & . & \sigma_{\beta 3}^{2} \end{array}\right]$$

and matrix V is defined as:

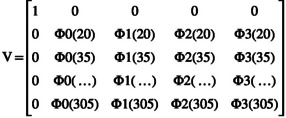

Heritability on each day of lactation (DIM) was calculated using the variance functions derived from the backward regression model, following Jamrozik et al. (1997), given by the following formula:

$$h^{2}\left(t\right)=\frac{\;z{\left(t\right)}^{\prime }\mathrm{Gz}\left(t\right)\;}{z{\left(t\right)}^{\prime }\mathrm{Gz}\left(t\right)+z{\left(t\right)}^{\prime }\mathrm{PEz}\left(t\right)+\mathrm{\sigma e}2}$$

where *z*(*t*) is the vector of Legendre polynomials evaluated at DIM *t*, and *G* and PE are the genetic and permanent environmental (co)variance matrices, respectively, σe^2^ is the residual variance. Heritability for a regression coefficient was calculated as the ratio of genetic variance over the sum of genetic, CG and permanent environmental variances for a respective component (Jamrozik et al. 2001).

The (co)variance components and the prediction of genetic parameters were estimated using Bayesian inference through GIBBS2F90 software (Misztal 2015). For each bivariate analysis, sampling chains of 500,000 iterations were generated, with an initial burn‐in of 50,000 iterations and a thinning interval of 20, resulting in a total of 22,500 samples for analysis. Convergence of the sampling chains was assessed using the Geweke (1992) criterion, with calculations performed via the BOA package in R software (Smith 2007).

## 3 Results

Descriptive statistics (Table 1) indicate that the estimated means for the traits evaluated are within the typical ranges observed in primiparous Holstein cows.

**FIGURE 1:**
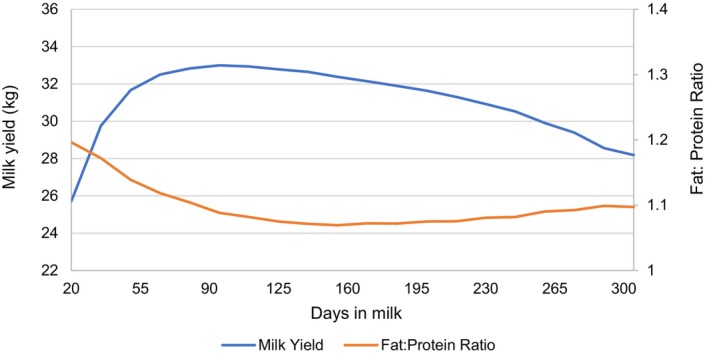
Average daily values of milk yield and fat: Protein ratio according to the days in lactation (fortnightly classes) in primary Holstein cows. [Colour figure can be viewed at wileyonlinelibrary.com]

Figure 1 illustrates the average TDMY and FPR curves throughout lactation in primiparous Holstein cows. TDMY ranged from 25.72 to 33.00 kg and, as expected, milk yield gradually increased until reaching a peak at 95 days in milk, where it attained 33.0 kg/day, followed by a continuous decline through the end of lactation.

The average FPR curve exhibited the opposite pattern compared to TDMY, ranging from 1.07 to 1.20 throughout lactation (Table S1). Higher values were observed immediately after calving, followed by a decreasing trend that stabilised between 1.07 and 1.08 from the 110th to the 245th day in milk. Thereafter, FPR experienced a continuous but slow increase until the end of lactation.

**FIGURE 2:**
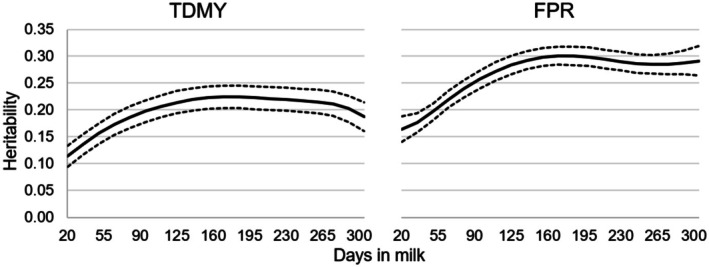
Posterior means (solid line—bold) and credibility interval (HPD = 95%) (dotted lines) for the heritability estimates of test‐day milk yield (TDMY) and fat: Protein ratio (FPR) as a function of days in milk (fortnightly classes) in Holstein cows.

Figure 2 presents the heritability estimates for TDMY and FPR. The genetic parameters obtained in this study for all traits were interpreted in accordance with the magnitudes established by Bourdon (2014, 169). Heritability estimates for TDMY ranged from 0.11 to 0.22, displaying low magnitude in early lactation. On the other hand, from the lactation peak at 95 days onward, the heritability estimates rose to moderate magnitudes (> 0.20). Heritability coefficients for FPR ranged between 0.16 and 0.30. Estimates up to 30 days in milk were of low magnitude, while after this period, the values increased, remaining moderate until the end of lactation.

Table 2 presents the heritability estimates of fertility traits. All traits were found to have low‐magnitude heritability.

**TABLE 2:** Heritability and credibility interval (HPD = 95%) of fertility traits in Holstein cattle from bivariate analyses.

Trait	h^2^
Period from calving to first service (CFS)	0.05 (0.03–0.07)
Days open (DO)	0.04 (0.02–0.05)
Calving interval (CI)	0.03 (0.02–0.05)

Abbreviations: h^2^ = heritability.

Table 3 shows the mean posterior estimates of heritabilities for and additive genetic correlations between the first two regression coefficients (a0 and a1) for TDMY and FPR and fertility traits (CFS, DO and CI). The estimated heritability for the first regression coefficient (a0), which corresponds to the overall production level during lactation, was 0.31 (0.01) for milk yield and 0.64 (0.01) for FPR. Heritabilities for persistency (a1 coefficient of TDMY and FPR) were 0.13 (0.01) and 0.23 (0.02), respectively, which are lower values than those estimated for the overall production level. Heritability estimates for the first regression coefficient (a0) of fertility traits were low, ranging from 0.04 (CFS and CI) to 0.07 (DO).

**TABLE 3:** Posterior means and credibility interval (HPD = 95%) of heritabilities (diagonal) and additive genetic correlations (above the diagonal) between the first two regression coefficients (a0 and a1) for milk yield, fat: protein ratio and fertility traits in Holstein cows.

Coef.	TDMY		FPR		CFS	DO	CI
	a0	a1	a0	a1	a0	a0	a0
**TDMY**							
a0	**0.31 (0.29–0.34)**	0.38 (0.28–0.48)	−0.31 (−0.36 to −0.26		0.25 (0.08 to 0.40)	0.22 (0.11 to 0.32)	0.32 (0.18 to 0.45)
a1		**0.13 (0.10–0.15)**		−0.25 (0.37 to −0.12)	0.18 (−0.11 to 0.37)	0.08 (−0.09 to 0.23)	0.09 (−0.10 to 0.29)
**FPR**							
a0			**0.64 (0.61 to 0.66)**	0.47 (0.36 to 0.52)	0.23 (0.11 to 0.35)	−0.11 (−0.18 to −0.05)	0.11 (0.00 to 0.23)
a1				**0.23 (0.19 to 0.27)**	−0.28 (−0.46 to −0.11)	−0.65 (−0.73 to −0.57)	−0.40 (−0.55 to 0.24)
**Fert.**							
CFS					**0.04 (0.03 to 0.06)**		
DO						**0.07 (0.05 to 0.09)**	
CI							**0.04 (0.02 to 0.05)**

Abbreviations: CFS = period from calving to first service, CI = calving interval, Coef. = coefficients, DO = days open, Fert. = fertility, FPR = fat: protein ratio, TDMY = test‐day milk yield.

Genetic correlations between the overall production level milk yield and fertility traits (Table 3) were positive and of moderate magnitude (a0 range: 0.22–0.32). Correlations between the persistency and fertility traits were positive but of low magnitude (a1 range: 0.08–0.18). Estimated genetic correlations between the overall production level of FPR (a0 coefficient) and CFS were moderate (0.23). The correlations between coefficient a0 of FPR with DO and CI were weak, at −0.11 and 0.11, respectively. In contrast, genetic correlations between the a1 coefficient of FPR and fertility traits were negative, varying from moderate to high (−0.28 to −0.65).

As also noted in Table 3, genetic correlations between the overall production level (a0) and persistency (a1) of milk yield and FPR were 0.38 (0.05) and 0.47 (0.04), respectively. The genetic correlation between the overall milk production level (a0) and FPR was negative (−0.31) and of moderate magnitude, similar to the correlation between milk persistency (a1) and FPR (−0.25).

**FIGURE 3:**
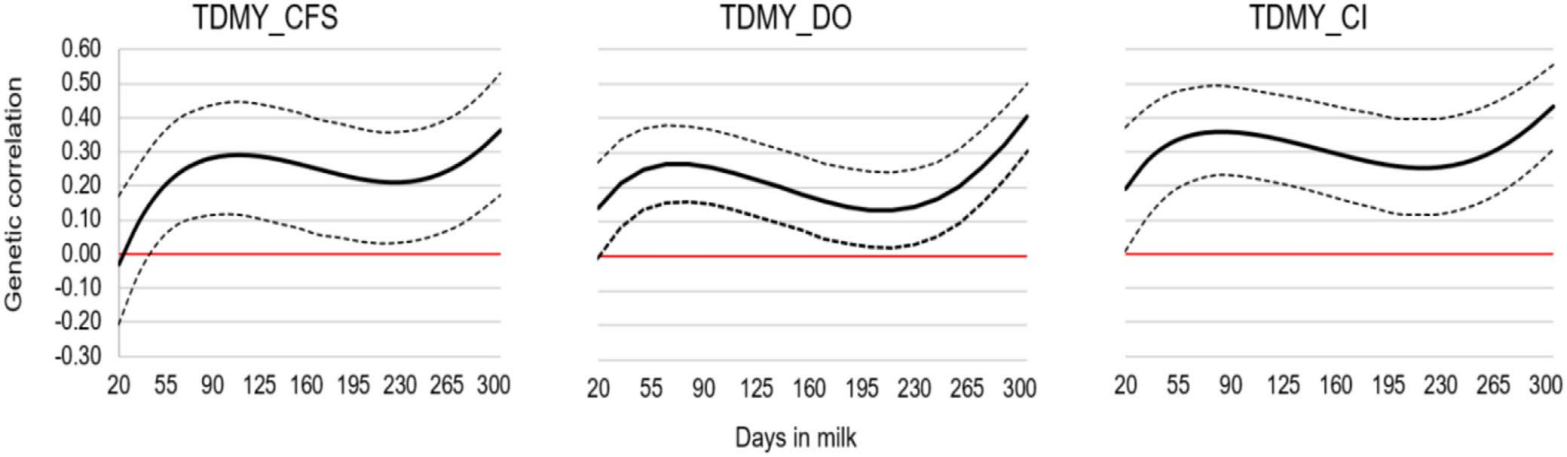
Posterior means (solid lines—bold) and credibility interval (HPD = 95%) (dotted lines) for the estimates of additive genetic correlations between TDMY and the period from calving to first service (CFS), days open (DO) and calving interval (CI) in first‐lactation Holstein cows. [Colour figure can be viewed at wileyonlinelibrary.com]

Figure 3 illustrates the genetic correlations between TDMY and fertility traits. Genetic correlations between TDMY and CFS exhibited a range from slightly negative to positive throughout lactation (−0.03 to 0.36). Conversely, genetic correlations between TDMY and DO, as well as between TDMY and CI, were consistently positive, ranging from 0.14 to 0.40 and from 0.19 to 0.43, respectively.

**FIGURE 4:**
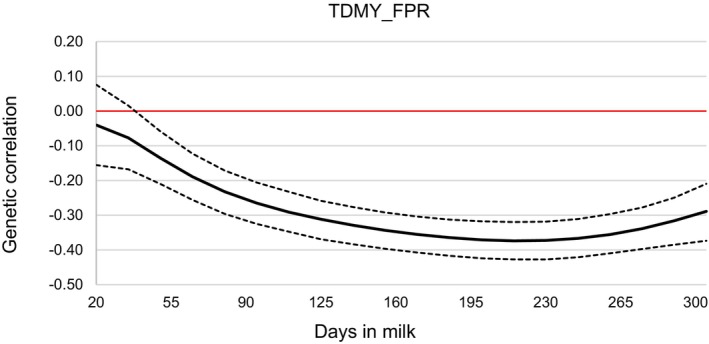
Posterior means (solid line—bold) and credibility interval (HPD = 95%) (dotted lines) for the estimates of additive genetic correlations between test‐day milk yield (TDMY) and fat: protein ratio (FPR) in first‐lactation Holstein cows. [Colour figure can be viewed at wileyonlinelibrary.com]

In this study, genetic correlation estimates between TDMY and FPR were negative throughout lactation, ranging from −0.04 on day 5 to −0.37 in the last third of lactation (Figure 4).

**FIGURE 5:**
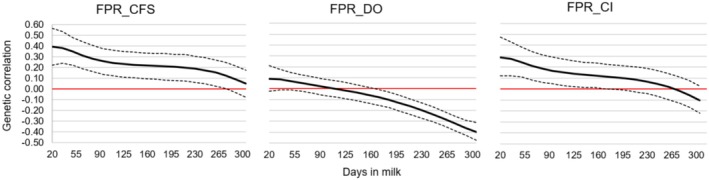
Posterior means (solid line—bold) and credibility interval (HPD = 95%) (dotted lines) for the estimates of additive genetic correlations between fat: protein ratio (FPR) and the period from calving to first service (CFS), days open (DO) and calving interval (CI) in first‐lactation Holstein cows. [Colour figure can be viewed at wileyonlinelibrary.com]

Genetic correlations between FPR and fertility traits displayed a declining trend during lactation, with positive values in early lactation (Figure 5). Genetic correlation estimates between FPR and CFS were positive throughout lactation, decreasing from 0.39 in early lactation to 0.05 at the end. Genetic correlations between FPR and DO ranged from low and positive to moderate and negative during lactation, gradually decreasing from 0.09 on day 20 to −0.40 on day 305. Similarly, genetic correlations between FPR and CI showed moderate and positive values in early lactation (0.29) transitioning to weak and negative by late lactation (−0.10).

## 4 Discussion

### 4.1 Descriptive Statistics and Production Curves

The average daily milk yield (31.20 ± 7.42 kg) was comparable to those reported by Carrari (2022) and Padilha et al. (2024) for Brazilian Holstein cow herds, which were 31.56 ± 9.94 and 32.24 ± 7.91 kg of milk, respectively. Additionally, both studies estimated FPR values ranging from 1.15 to 1.19, which are slightly higher than the average value of 1.10 found in the present study. However, the FPR value generally falls within the ideal range of 1.0–1.5, recommended in the literature (Enemark 2008); values outside this threshold indicate metabolic problems that could lead to reductions in milk yield and fertility.

Descriptive statistics for fertility traits were similar to those reported in previous studies with Holstein cow herds in Sweden, Canada and China by Tarekegn et al. (2019), Oliveira et al. (2021) and Hu et al. (2023), respectively. Despite intense selection for fertility traits in Holstein cattle, they warrant careful consideration in breeding programmes due to their potential impact on other reproductive and productive traits (Almeida 2017).

The mean milk yield curves presented in this study (Figure 1) followed a typical lactation curve shape, comparable to values reported by Siewert et al. (2019) and Innes et al. (2024). Both studies assessed first‐lactation Holstein cows, noting peak milk yields around the third month of lactation at 32.2 and 32.6 kg per day, respectively. Our results confirm a biologically normal lactation curve, with first‐lactation cows exhibiting later peaks and lower milk production compared to multiparous cows.

Throughout lactation, FPR means consistently remained within the ideal range of 1.0–1.5 (Enemark 2008). Gantner et al. (2016) suggest that an FPR below 1.0 may indicate a risk of ruminal acidosis, while values above 1.5 could signal susceptibility to ketosis, energy deficiency and other metabolic diseases (Koeck et al. 2013; Cabezas‐Garcia 2021). These findings reveal that cows in Paraná herds maintain metabolic balance (Padilha et al. 2024), potentially implying an adaptation to NEB.

### 4.2 Heritability for Daily Yield and Fertility Traits

Random regression models allow for the estimation of heritability coefficients for longitudinal traits, such as milk yield and FPR, across different stages of lactation, offering significant advantages for interpreting these estimates (El Faro and Albuquerque 2003).

In this study, heritability estimates (Figure 2) demonstrated a substantial environmental influence on milk yield in early lactation. Similar trends in daily heritability were reported by Paiva et al. (2022), who observed lower coefficients for milk yield during this period. Guinan et al. (2024) noted that low heritability estimates during this time result from large residual variances and the fact that early lactation is the most challenging period for dairy cows.

However, from the middle to the end of lactation, there appears to be larger potential for genetic gain in milk yield through selection. Naderi (2018) and Soumri et al. (2023) estimated heritability coefficients for TDMY ranging from 0.11 to 0.22 throughout lactation, findings that align with those of this study. Conversely, Wahinya et al. (2020) estimated higher heritability coefficients (0.21–0.60) across the entire lactation curve, suggesting that genetic gains for TDMY are achievable through selection.

The heritability estimates for FPR in early lactation obtained in this study (Figure 2) are comparable to those reported by Koeck et al. (2014) and Benedet (2020), with values ranging from 0.08 to 0.14 between 5 and 35 days in milk. Negussie et al. (2008) stated that high environmental variation might contribute to the lower heritability of FPR during early lactation. Padilha et al. (2024) noted that the relatively low heritability estimates for FPR in early lactation could be associated with selection emphasis on higher initial yield milk, which may mask the genetic variation for FPR.

Throughout lactation, the heritability coefficients for FPR were moderate (> 0.20), indicating that direct selection for this trait during the entire lactation may yield genetic gains and could therefore be included among the selection criteria for the Holstein breed in Southern Brazil. This is supported by Jamrozik and Schaeffer (2012), who reported that FPR is highly heritable and could serve as an additional indicator for indirect selection against metabolic diseases in dairy cattle.

The estimates obtained in the present study were higher than those reported by Buaban (2016), who found heritabilities ranging from 0.17 to 0.19 throughout lactation using random regression models. They concluded that FPR could be utilised as a selection criterion to enhance fertility traits and milk yield, and should thus be considered in breeding programmes and selection indices with appropriate economic weights.

The estimated heritability coefficients for fertility traits (Table 2) suggest a strong influence of non‐genetic factors on their expression. As such, genetic gains through direct selection will be small in each generation. Improvements in environmental factors, such as heat detection techniques, training in insemination practices and herd management, may yield more significant enhancements in these traits.

Overall, heritability estimates for fertility traits (Table 2) were similar to those reported in studies conducted in several countries with Holstein animals (Albarrán‐Portillo and Pollott 2013; Almeida 2017; Tarekegn et al. 2019; Alves 2020; Oliveira et al. 2021; Kgari et al. 2022). The authors concluded that fertility traits are greatly influenced by environmental factors and that direct selection for these traits will result in slow genetic progress, supporting the hypothesis that fertility traits are difficult to use directly as selection criteria (Almeida 2017).

In Brazil, one of the selection indices used for the Holstein breed, the ISG/PR (Genetic Selection Index—Paraná), assigns a weight of 12% to the health and fertility component (APCBRH 2024). Due to the modest weighting of fertility traits in the ISG/PR and their low heritabilities, Oliveira et al. (2021) emphasised that dairy producers should not expect rapid genetic improvements for these traits in the short term. Nonetheless, these traits are crucial for determining the reproductive efficiency of the herd (Ali et al. 2019).

### 4.3 Genetic Parameters for Overall Milk Production Level, FPR and Persistency

Table 3 presents the mean posterior estimates of heritabilities and genetic correlations for the first two regression coefficients (a0 and a1) for productive and fertility traits. The first Legendre polynomial coefficient (a0), associated with the additive genetic effect, is related to the overall production level during lactation. In contrast, the second coefficient (a1) relates to lactation persistency. According to Jamrozik et al. (2002), this distinction is because the eigenvector matrices for genetic and permanent environmental components are nearly diagonal, with weights corresponding to the original variables in Legendre polynomials. This setup describes an overall production level in lactation (a0) and a persistency component (a1).

The heritability estimates for a0, representing the overall production level up to 305 days for milk yield and FPR, were higher than those estimated for persistency (coefficient a1). Pereira et al. (2013) observed similar behaviour for milk, fat and protein yield in dairy Gir cows. However, in our study, the heritability estimates for a0 of fertility traits confirm that environmental factors significantly influence the performance of these traits in Holstein cows. Thus, the use of high‐quality semen and enhanced inseminator skills can substantially improve these traits.

Genetic correlations between a0 and a1 of milk yield and fertility traits (Table 3) revealed that higher total milk yield and better lactation persistency are genetically associated with reduced fertility, characterising an unfavourable association. Yamazaki et al. (2014) reported a similar finding in Holstein cows in Japan, estimating genetic correlations of 0.17 ± 0.04 and 0.20 ± 0.04 between a1 and CFS, and a1 and DO, respectively. The authors highlighted that as increases in milk volume and persistency are observed, fertility indicators should be incorporated into genetic evaluations to mitigate undesirable effects on cow fertility.

The genetic correlation between a1 and CI (0.09) was found to be similar in magnitude to that reported by Muir et al. (2004) for Holstein cattle in Canada, which was 0.17 ± 0.09. That study noted that greater persistency in the first lactation correlates with a longer interval between the first and second calvings, which is typically considered undesirable due to the antagonistic genetic association between persistency and CI in the first lactation. According to Cesarani (2020), the relationship between persistency and CI might be influenced by milk yield.

It is important to recognise that in addition to sample effects, the varying definitions of persistency used across different studies can significantly affect estimates. This is because the association between persistency and other traits, such as milk yield and fertility, depends on the method used to define and estimate persistency (Pereira et al. 2012). A persistency metric less correlated with yield will likely provide different correlation estimates with fertility traits, given the unfavourable genetic association between yield and fertility.

The genetic correlations found between a0 of FPR and fertility traits (Table 3) suggest that dairy cows with a higher overall production level for FPR tend to have longer CFS, while the associations with DO and CI indicate that selection for one of these traits would not significantly affect the others. Conversely, selecting cows in the first lactation that exhibit higher a1 for FPR would shorten the CFS, DO and CI intervals, which are desirable.

The genetic correlation between a0 and a1 of both yield traits (milk and FPR) shows that selecting for increased persistency would enhance total milk volume and FPR. However, these estimates should be of low magnitude to justify their inclusion in genetic improvement programmes. If the genetic correlation were negative, selection for persistency could adversely affect genetic gains for milk yield (Pereira et al. 2012).

Our results differ from those obtained by Pereira et al. (2013), who estimated a negative and moderate correlation (−0.21) between the first two regression coefficients for milk yield in a herd of Gir cows. The disparities between these studies can be attributed to variations in population samples, breed characteristics, management conditions and the production levels of the herds studied. In Holstein cows, for instance, the milk production level is higher and the peak production occurs later compared to the Gir breed. For FPR, the genetic correlation between a0 and a1 indicates that these traits are largely influenced by the same sets of genes.

The estimated genetic correlation between the overall milk production level (a0) and FPR demonstrates that as milk yield increases, there is a tendency for the proportion of milk solids (fat and protein) to change, potentially influencing FPR, a behaviour expected due to the dilution effect. A similar correlation was reported by Jamrozik and Schaeffer (2012), who found a value of −0.30 (±0.02) in Holstein cows in Canada.

Additionally, the genetic correlation between milk a1 and FPR was negative and of moderate magnitude, denoting an antagonistic relationship between them. It is important to note that the FPR has received relatively less attention in genetic studies of dairy cattle, and estimates of genetic parameters for regression coefficients for this trait are scarce. To date, no published studies have specifically addressed these estimates for fertility traits.

### 4.4 Genetic Correlations Between Milk Yield and Fertility Traits

Figure 3, which presents the genetic correlations between TDMY and fertility traits, reveals that the values of these correlations were generally unfavourable. Buaban (2016) estimated unfavourable genetic correlations between TDMY and fertility traits (CFS, DO and CI), indicating that selection for increased milk yield is associated with longer CFS, DO and CI periods. Similarly, Puangdee et al. (2017) observed that in first‐lactation Holstein cows, genetic correlations between TDMY and DO increased throughout lactation and concluded that selection for high milk yield will result in increased DO.

This suggests that selection for increased milk yield, especially around the peak and end of lactation, could lead to longer periods (in days) from the time of calving to the first insemination (CFS), from calving to conception (DO) and a longer CI, all of which are undesirable outcomes.

### 4.5 Genetic Correlations Between Milk Yield and FPR

Understanding the genetic correlations between TDMY and FPR has been important for improving FPR as an indicator of energy balance, especially since primiparous cows with high milk production early in lactation often experience poor metabolic states (Nishiura et al. 2015). The FPR is valuable in selection for both milk yield and fertility within the Holstein breed (Puangdee et al. 2017).

In this study, genetic correlations between TDMY and FPR (Figure 4) during early lactation (up to 35 days) were low, ranging from −0.04 to −0.08, meaning that selecting for one trait would have minimal impact on the other. Nishiura et al. (2015) similarly reported weak correlations, implying that reducing FPR would not significantly affect milk yield.

Conversely, Negussie et al. (2013) estimated positive genetic correlations (0–0.21) between TDMY and FPR in early lactation (up to 65 days), suggesting that high‐yielding cows mobilise body reserves to meet energy demands, leading to higher FPR. However, after peak lactation, these correlations declined to near zero or negative, possibly indicating recovery from NEB.

The negative correlation between TDMY and FPR estimated in our study (−0.04 to −0.19, from 20 to 65 days in milk) demonstrate that high‐producing Holsteins may not be experiencing severe NEB. This aligns with the highly technological and nutritionally supported production systems represented in our dataset. Supporting this, Jamrozik and Schaeffer (2012) found negative genetic correlations between TDMY and FPR in first‐lactation cows in Canada, reinforcing FPR's potential as an indirect selection tool for mastitis. Similarly, Sungkhapreecha et al. (2022) found stronger negative genetic correlations using BLUP (−0.21) and ssGBLUP (−0.18) in Holsteins in Thailand, suggesting that high‐yielding cows in those systems were more susceptible to NEB.

From mid to late lactation, we found negative and moderate genetic correlations between TDMY and FPR particularly around peak milk production (80–110 days, −0.23 to −0.29) and in the final third of lactation (−0.29 to −0.37). These findings indicate that selection for milk yield in these periods may contribute to a more favourable (lower) FPR.

Likewise, Puangdee et al. (2017) observed a similar decrease in the genetic correlation between 305‐day milk yield (TDMY) and FPR throughout lactation, from zero on day 5 to −0.46 on day 305, emphasising the negative impact of a high FPR on milk yield, especially in late lactation. Similarly, Satola and Ptak (2019) estimated consistently negative correlations (−0.30 to −0.62) throughout the first lactation, which shows that reducing FPR in early lactation could positively influence overall milk production.

Thus, given the weak genetic correlations between FPR and TDMY in early lactation, selection for milk yield would likely have minimal effects on FPR. However, selection around peak and late lactation could be strategically beneficial in mitigating metabolic disturbances and optimising production efficiency.

### 4.6 Genetic Correlations Between FPR and Fertility Traits

The energy imbalance resulting from NEB, observed primarily in the early phases of lactation, can directly influence the reproductive performance of dairy cows (Padilha et al. 2024). Determining the genetic correlations between FPR and fertility traits is of fundamental importance for dairy cattle, aiming to better elucidate the relationship between these traits and the selection of robust animals capable of overcoming metabolic challenges in early lactation.

Genetic correlations between FPR and fertility traits are depicted in Figure 5. In this study, the estimates of genetic correlation between FPR and CFS in early lactation (0.31–0.39) suggest that selecting for reduced CFS will lead to a lower FPR during this period. Mehtio et al. (2020), evaluating Nordic Red herds, reported positive correlation values between FPR and CFS that also decreased throughout lactation, recording 0.18 (between 8 and 35 days), 0.03 (between 36 and 63 days) and 0.01 (between 64 and 91 days). They highlighted that prolonged CFS is often due to NEB.

Negussie et al. (2008) found positive and moderate correlations between FPR and CFS in early lactation (0.28), with negative and low correlations at the end of lactation (−0.04), indicating a similar tendency for these values to decrease as lactation progresses. The authors stated that this pattern is expected, as NEB affects the period between calving and the onset of ovarian activity. Therefore, CFS is closely linked to luteal activity, as it encompasses the interval from calving to the first insemination.

In contrast, the positive but low‐magnitude genetic correlations between FPR and DO (0.02–0.09) signify that these traits are weakly associated during the first third of lactation. In the final third of lactation, negative estimates of moderate magnitude (−0.20 to −0.40) imply an unfavourable genetic relationship between FPR and DO. This finding means that high FPR levels in cows during this phase may be linked to a reduced calving‐to‐conception interval.

The genetic correlations between FPR and CI in early lactation observed in this study indicate that selecting for a lower FPR may reduce CI, which is generally beneficial. Nonetheless, extremely low FPR values can predispose cows to ruminal acidosis (Gantner et al. 2016). Padilha et al. (2024) observed a positive and moderate genetic correlation (ranging from 0.20 to 0.31) between FPR and CI in Holstein cows, noting that a reduced CI could lead to lower FPR. They also stressed the importance of maintaining an optimal FPR range to balance fertility traits, particularly in the postpartum period.

In the mid and late stages of lactation, the genetic correlation between FPR and CI suggests limited shared genetic control, indicating that selection for one trait would have little effect on the other. High FPR in early lactation likely reflects the negative effects of NEB, which may impair fertility traits such as calving‐to‐first‐service interval (CFS) and CI, leading to longer reproductive intervals. Moreover, elevated FPR may be associated with NEB‐related metabolic disorders.

## 5 Conclusions

Heritability estimates for milk yield and FPR support the potential for genetic improvement of these traits, whereas the low heritability of fertility traits underscores the limitations of direct selection for reproductive performance. Although increasing milk yield remains a primary breeding objective, prioritising production near peak lactation may improve total yield but can also negatively affect fertility. This strategy often results in high FPR during early lactation, likely reflecting the adverse effects of NEB, which can impair reproductive traits.

Alternatively, selecting for more persistent lactation curves—with smaller differences between peak and late‐lactation yields—may offer a more balanced approach. This strategy could help optimise both milk production and fertility outcomes. Furthermore, the observed associations between FPR and reproductive traits suggest that elevated FPR values in early lactation may hinder conception, while higher values later in lactation may be linked to shorter open periods.

## Conflicts of Interest

The authors declare no conflicts of interest.

## Supporting information


Data S1.

## Data Availability

The datasets generated and/or analysed during the current study are available from the corresponding author upon reasonable request and with the authorisation of APCBRH.
