# Supplementary material for: Genetic Insights: Balancing Milk Yield, Fat: Protein Ratio and Fertility in Primiparous Cows From Subtropical Regions
Source: J Anim Breed Genet. 2025 May 30;142(6):753–64. doi: 10.1111/jbg.12944 (PMC12501733; doi:10.1111/jbg.12944)
Supplement: Supplementary file 1 — Data S1. [file JBG-142-753-s001.docx]

**Table S1** - Descriptive statistical summary of FPR values and their standard deviation across DIM classes in first-lactation Holstein cow.

| DIM | N | FPR | Standard Deviation |
| --- | --- | --- | --- |
| 20 | 20934 | 1.196263 | 0.2193644 |
| 35 | 22506 | 1.172138 | 0.2086441 |
| 50 | 22410 | 1.138891 | 0.2079644 |
| 65 | 21870 | 1.118419 | 0.2059535 |
| 80 | 22198 | 1.104244 | 0.2101942 |
| 95 | 22029 | 1.088348 | 0.2109542 |
| 110 | 22337 | 1.081988 | 0.2106068 |
| 125 | 21958 | 1.07501 | 0.2125467 |
| 140 | 22500 | 1.071392 | 0.2120119 |
| 155 | 21958 | 1.069338 | 0.2119845 |
| 170 | 22292 | 1.072266 | 0.2121946 |
| 185 | 21789 | 1.071973 | 0.2095338 |
| 200 | 22074 | 1.075083 | 0.2098013 |
| 215 | 21480 | 1.075395 | 0.2076601 |
| 230 | 21789 | 1.080882 | 0.2033506 |
| 245 | 21330 | 1.081842 | 0.2047146 |
| 260 | 21256 | 1.090344 | 0.2035954 |
| 275 | 19389 | 1.092607 | 0.200573 |
| 290 | 12609 | 1.098926 | 0.1994394 |
| 305 | 9092 | 1.097173 | 0.1966462 |

N: Number of observations; FPR: Fat-to-Protein Ratio
